# Internet analytics of an innovative digital educational resource of type 1 diabetes HelloType1 in local languages for people living with diabetes families and healthcare professionals in Southeast Asia

**DOI:** 10.1186/s12902-023-01501-4

**Published:** 2023-11-16

**Authors:** Sze May Ng, IV Malene, Thy Khue Nguyen, Khuong Le, Yang Xian Lucas Lim, Ngee Lek, Samantha Seal, Steffen Yun Tange, Azriyanti Anuar Zaini, Taninee Sahakitrungruang, Anne Charlotte Ficheroulle

**Affiliations:** 1 University of Liverpool and Edge Hill University, Liverpool, UK; 2 Paediatric Department, Mersey and West Lancashire Teaching Hospitals NHS Trust, Ormskirk, L39 2AZ UK; 3 Kantha Bopha Children’s Hospital, Phnom Penh, Cambodia; 4 Ho Chi Minh City University of Medicine and Pharmacy, Ho Chi Minh Medical Association, Ho Chi Minh, Vietnam; 5 University of Social Sciences and Humanities, Vietnam National University, Ho Chi Minh City, Vietnam; 6 Dietitian 90 Consultancy, Georgetown, Malaysia; 7 KK Women’s and Children’s Hospital, Singapore, Singapore; 8 T1D Advocate, Bangkok, Thailand; 9 Summit Consulting, Copenhagen, Denmark; 10 University Malaya, Kuala Lumpur, Malaysia; 11 School of Global Health, Faculty of Medicine, Chulalongkorn University, Bangkok, Thailand; 12 Action 4 Diabetes, Somerset, UK

**Keywords:** Action4diabetes, Hellotype1, Diabetes, Education, Digital learning, Southeast Asia

## Abstract

**Background:**

There is minimal data of health outcomes for Type 1 Diabetes (T1D) in Southeast Asia (SEA) where government funding of insulin and blood glucose monitoring either do not exist or is limited. The full impact of Covid-19 pandemic on the national economies of SEA remain unknown. In the midst of the pandemic, in 2021, HelloType1 was developed by Action4Diabetes (A4D), a non-government organisation charity in collaboration with Southeast Asia local healthcare professionals as an innovative digital educational resource platform of T1D in local languages. HelloType1 was launched in Cambodia, Vietnam, Thailand and Malaysia in 2021 to 2022 with Memorandums of Understandings (MOUs) signed between A4D and each country. Internet data analytics were undertaken between the 1^st^ of January 2022 to 31^st^ of December 2022.

**Aims:**

The aims of this study were to explore the usability and internet data analytics of the HelloType1 online educational platform within each country.

**Methods:**

The data analytics were extracted Google analytics that tracks data from the website hellotype1.com and Facebook analytics associated with the website.

**Results:**

There was a 147% increase in the number of HelloType1 users between the first 6 months versus the latter 6 months in 2022 and a 15% increase in the number of pages visited were noted. The majority of traffic source were coming from organic searches with a significant increase of 80% growth in 2022.

**Conclusions:**

The results of the analytics provide important insights on how an innovative diabetes digital educational resource in local languages may be optimally delivered in low-middle income countries with limited resources.

## Introduction

Action4Diabetes (A4D) is a United Kingdom based non-government organisation (NGO) charity formed in 2016 that is making sustainable and scalable progress to provide quality Type 1 diabetes (T1D) healthcare in the Southeast Asia (SEA). A4D currently provides comprehensive partnership programmes with defined local hospitals through a Memorandum of Understanding (MOU) signed with the governments in SEA that guarantees ongoing supplies of free insulin, blood glucose testing, HbA1c tests and hospital emergency funds in in low-middle-income countries (LMICs)—Laos, Malaysia, Vietnam, Cambodia, Thailand and Myanmar [1, 2]. It is noted that there is no published data of prevalence and incidence of T1D in these countries [1]. There is limited availability of diabetes information in local languages for people with T1D and families living with this chronic condition in LMICs in SEA. The conventional approach of publishing leaflets and distributing through clinics, although done with the best of intentions, this outreach of information is limited.

Many people with T1D in SEA live hundreds of miles from hospitals and the conventional approach of T1D education in clinics have a limited outreach. Internet access in SEA has boomed in the last decade and is now more affordable with cheaper phones and data access. Because of this, digital access is now the most efficient solution to reach out, educate and engage with the T1D community. Digitalisation of healthcare has enabled health education to be available to a wider audience and in a more accessible manner [3]. In chronic conditions like diabetes, management of children, families and carers can be complex and challenging [4]. Promoting an online educational environment requires an integrated approach with healthcare professionals, and patient education improves patient understanding and adherence to medical instructions, which has a positive impact for those with chronic health conditions [5, 6].

In 2021, during the pandemic peak, A4D developed HelloType1 as an innovative digital healthcare educational resource platform of T1D in local languages for families and people with T1D in collaboration with SEA local healthcare professionals. HelloType1 aims to educate, engage and empower healthcare professionals, families, carers and people with T1D by creating a digital platform that is free-to-access and provides accurate up-to-date information, patient-oriented education and best practice about T1D care in the local languages of each country. The objectives of this study were to explore the usability and internet data analytics of the HelloType1 platform within each country.

The advent of rapid technological advancement, mobile phone devices and ease of access to the internet has drastically changed societies, even in low-middle income countries like SEA. Digitalisation of education and healthcare has been considered a time-effective and cost-effective alternative to traditional patient education [7]. Positive impact have also been reported with internet-based and computer-enhanced education, including educational video [8]. Digital teaching and learning gained increasing attention particularly during the pandemic when travel ceased. The target audience for digital education amongst health professionals have now evolved to include students, patients and their caregivers [9]. Building a digital health education and resource is an area that is inevitable and will be a part of a global health care education in the future [10]. These will include adoption of digital health resources into easy-to-use platforms for patients and caregivers using multiple approaches, though each with a challenge to consider.

In this era, we will face challenges with technology advancements and integration of digital education into a curriculum. A generational shift is observed where the new Gen Z (people born after the year 1997) are considered as “digital natives” in accepting the use of technology in their lives. As such, the clinical implications of digitalisation of healthcare and education for patients will force developments of innovative methods to engage users, and to educate and introduce new topics to learners. Although opportunities are plentiful, faculty development and experts to facilitate learning experiences may not be available at the same scale [10].

## Methods

The HelloType1 programme comprises of a website designed as an educational resource centre for T1D information in local languages and local language Facebook pages aimed to engage and empower patients and carers. The HelloType1 website has been designed for 2 core audiences. A user option tab of choosing either ‘healthcare professional’ or ‘people living with T1D and caregivers’ on entry to the website. It is free to access and covers eight core topics. Content is curated for the topics using information taken from accredited sources such as the International Diabetes Foundation (IDF) and the International Society for Paediatric and Adolescent Diabetes (ISPAD). All content is reviewed, translated to local languages and adapted towards the SEA culture by a panel of local and international T1D healthcare experts and lay people with T1D in SEA to ensure that the information is appropriate in a local context. A list of sources and content reviewers are published within each core topic. The content has been tailored to each audience needs in different format for more effective learning. For patients and caregivers, the content developed include 36 articles, 8 posters, 13 animated videos, 2 patient stories and 13 quizzes. For healthcare professionals, they can access a list of curated international resources, webinar series on T1D management and participate in regional network meetings.

HelloType1 was launched in English and Khmer languages in March 2021, in Vietnamese language in March 2022, in Thai language in November 2022 and in Bahasa (Malaysia) language in March 2023. A HelloType1 Facebook page was also created for each country in their local languages for sign-posting to the HelloType1 website.

For each country, a Facebook page has been created in local language in order to engage and empower patients and caregivers. The content posted is a mix of educational content shared from HelloType1 website and inspirational content to provide psychosocial support to help the local T1D community overcoming their struggles and stigma linked to T1D. It includes also online workshops hosted on zoom and broadcasts through Facebook live, allowing patients and caregivers to interact and get support from local experts or advocates.

A4D signed Memorandums of Understandings (MOUs) with the Cambodian Diabetes Association (CDA), the Vietnamese Association of Diabetes and Endocrinology (VADE), the Diabetes Association of Thailand (DAT), the Malaysian Endocrine and Metabolic Society (MEMS), the Malaysian Diabetes Educator Society (MDES) and the Myanmar Society of Endocrine and Metabolism (MSEM) to have the HelloType1 content ratified and endorsed locally before official publication and launch in these countries.

We undertook internet data analytics between the 1^st^ of January 2022 to 31^st^ of December 2022. Comparisons are made within each quarter of the annual year for the HelloType1 website and the HelloType1 Facebook sites within each country. Facebook definitions include the following, followers are defined as number of Facebook users who are following the HelloType1 page, ‘Reach’ is defined as the number of times a post from the HelloType1 page appear on a Facebook user’s feed, ‘Engagement rate’ is defined by the % of people interacting with a content posted on the page via total number of shares, likes, comments divided by the total post reach, and ‘Click-through-rate’ (CTR) is defined as the % of people clicking on a Facebook link divided by the total post reach. All HelloType1 Facebook Pages are managed 100% organically with no budget spent to increase reach. Two posts are published every week on each. The term organic search refers to traffic source coming from search engines for example searching for information in local languages using the most common keywords: “sugar”, “glucose levels”, “blood sugar levels” “diabetes”.

The data analytics were extracted from the platform Google analytics that tracks data from the website www.hellotype1.com. Data from the Facebook analytics were extracted from tracking all user interaction and user behaviour related to the HelloType1 local Facebook page. These data analytic tools enabled us to extract the data by locality, gender, age, type of device used and how the user interacts with the content.

## Results

There has been a 147% increase in the number of users between Q1/Q2 versus Q3/Q4 in 2022 and a 15% increase in the number of pages visited (pageviews). An average of 2 pageviews per session were noted (Table 1). Our biggest users are the Cambodian community with more 1,400 followers. Table 2 shows that majority of traffic source were coming from organic searches with a significant increase of 80% growth from month to month in 2022. Organic searches refers to users actively looking for diabetes information online in their local languages. Table 3 shows that the majority of pageviews of content were coming from healthcare from families and people living with T1D.

**Table 1:** Overview analytics HelloType1 website (May 2021-Dec 2022)

	**2021**		**2022**			
	**Q3**	**Q4**	**Q1**	**Q2**	**Q3**	**Q4**
**Total users by country**	**408**	**736**	**826**	**2 839**	**2 640**	**2 808**
Cambodia			350	850	1 525	1 435
Vietnam			100	586	409	423
Thailand			22	81	77	288
Malaysia			25	149	41	46
Other countries			329	1 173	588	616
**Total pageviews per languages**	**902**	**1 922**	**2 579**	**9 299**	**5 315**	**8 399**
English		1 301	993	4 555	1 553	1 461
Khmer		621	741	1 160	2 295	2 744
Vietnamese			845	3 584	1 467	2 837
Thai						1 357
**Average time spent/article (seconds)**						
English			84	142	161	217
Khmer			242	208	214	300
Vietnamese			71	250	207	205
Thai						205

User; number of unique users visiting the website, Pageviews; number of pages visited on the website

**Table 2:** Traffic source of users

	**2022**			
	**Q1**	**Q2**	**Q3**	**Q4**
**Traffic source**				
Direct to website	26%	42%	16%	14%
Social Media eg via Facebook, Twitter	35%	15%	14%	15%
Organic search	35%	35%	62%	66%
Referral via link from other websites	4%	8%	8%	5%

**Table 3:** Pageviews of content being viewed- Average per quarter 2022

	**2022**			
	**Q1**	**Q2**	**Q3**	**Q4**
**Which content is being viewed**				
Information for People living with T1D	20%	37%	47%	42%
Information for Family Caregivers	18%	11%	21%	23%
Information for Healthcare professionals	10%	12%	6%	5%
Other content ie homepage	52%	40%	26%	30%

Table 4 shows the number of followers from the Facebook sites, the engagement rate and CTR stratified by country. Number of followers between the first 6 months and the latter 6 months of 2022 showed an organic growth of 27%, and 70% in the average monthly reach. In Q1/2 of 2022, most of the website pageviews visited was driven by the homepages (approx. 46%) which demonstrate a “discovering” phase of the user. In Q3/Q4, 45% of the website is driven by the pageviews in the ‘People living with T1D category’ and 22% by the ‘Family and Caregivers category’ which show that users are now used to the website and use it properly for its purpose. The ‘Healthcare Professionals category’ was often linked to specific events and the traffic source is direct for example when there is a new webinar or event for that is shared directly with them by email and they link to the website through this invitation. Demographics of HelloType1 followers from Vietnam and Cambodia are mostly from young people living with T1D between 18–34 years. The second biggest age category are T1D caregivers between the ages of 35–44 years as shown in Table 5 and 6. Table 7 and 8 show the healthcare professionals registered by profession and by country.

**Table 4:** Facebook performance 2022

	**2022**			
	**Q1**	**Q2**	**Q3**	**Q4**
**Nb Followers**				
KH	962	1 059	1 207	1 238
VN	45	111	165	178
TH				70
***Total***	**1 007**	**1 170**	**1 372**	**1 486**
**Av. Monthly growth**				
KH		10%	14%	3%
VN		147%	49%	8%
TH				
***Total***		**16%**	**17%**	**8%**
**Av. Monthly Post Reach**				
KH	2 756	4 545	6 737	4 231
VN	906	931	1 291	1 136
TH				2 984
***Total***	**3 662**	**5 476**	**8 028**	**8 351**
**Av. Links CTR**				
*KH*			7%	6%
*VN*		5%	10%	15%
*TH*				6%
***Total***		**5%**	**9%**	**9%**
**Av. Engagement rate**				
*KH*			10%	10%
*VN*		3%	3%	5%
*TH*				5%
***Total***		**3%**	**6%**	**6%**
**Av. Monthly Page visit**				
*KH*	89	154	348	371
*VN*	174	166	162	141
*TH*				388
***Total***	**263**	**320**	**510**	**900**

Followers; number of Facebook users who are following the HelloType1 page, reach; defined as the number of times a post from the HelloType1 page appear on a Facebook user’s feed, engagement rate; defined by the % of people interacting with a content posted on the page via total number of shares, likes, comments divided by the total post reach, click-through-rate (CTR); defined as the % of people clicking on a Facebook link divided by the total post reach

Facebook page Khmer (KH): https://www.facebook.com/HelloType1cambodia

Facebook page Vietnamese (VN): https://www.facebook.com/HelloType1vietnam

Facebook page Thai (TH): https://www.facebook.com/HelloType1Thailand/

**Table 5:** Facebook demographics Cambodia

Gender/Age	18–24	25–34	35–44	45 +	Total
Woman	11%	29%	13%	2%	55%
Man	9%	21%	11%	4%	45%
	**20%**	**50%**	**24%**	**6%**

**Table 6:** Facebook demographics Vietnam

Gender/Age	18–24	25–34	35–44	45 +	Total
Woman	10%	41%	14%	4%	69%
Man	2%	17%	7%	5%	31%
	**12%**	**58%**	**21%**	**9%**

**Table 7:** Healthcare professionals registered on HelloType1 by profession

Profession	%
Doctor	68%
Nurse	12%
Dietician	6%
Pharmacist	2%
Other HCP	12%

**Table 8:** Healthcare professionals registered on HelloType1 by country

Country	%
Cambodia	4%
Malaysia	2%
Myanmar	6%
Thailand	28%
Vietnam	40%
Outside of SEA	20%

### Cambodia

The HelloType1 Cambodia Facebook analytics showed approximately 1200 followers and it had a 4% increase each month. On average 2 to 3 posts are published every week which generates a monthly average reach of 4000. Cambodian users represent 56% of the total users but only approximatively 50% of the total pageviews are viewed in Khmer. 38% of the total pageviews viewed in Khmer are articles and users are spending on average 235 s per article. 70% of the followers are under 34 years old and 55% of followers are women. Further analytics show that one follower in Cambodia generates 4 reach on average, with an engagement rate of 10% and a CTR at 6%.

### Vietnam

Vietnamese users represent 18% of the total users and approximatively 35% of the total pageviews are viewed in Vietnamese. Most users are people living with T1D or family caregivers. 28% of the total pageviews viewed in Vietnamese are articles and users are spending on average 192 s per article. In Vietnam, 70% of the followers are women and are below 34 years of age. Further analytics show that one follower in Vietnam generates 8 reach on average, with an engagement rate of 4% and a CTR at 13%.

## Discussion

Diabetes education is a key factor within Type 1 diabetes management and has been shown to lead to better outcomes [11]. Delivery of diabetes education is a complex intervention which aims to provide the person and family/carers living with diabetes the knowledge and skills to successfully self-manage diabetes [12]. In the International Diabetes Federation Atlas, 10^th^ edition, there are an estimated 90 million type 1 diabetes people living in Southeast Asia rising to 152 million in 2045. To date, no registries exist in the region. Findings of the current 10th edition confirm that diabetes is one of the fastest growing global health emergencies of the twenty-first century while the number of children and young people up to 19 years old living with diabetes has been increasing each year. In 2021, over 1.2 million children and adolescents have type 1 diabetes [13].

The results of the analytics provide important insights regarding how an innovative diabetes digital educational resource in local languages may be optimally delivered in low-middle income countries with limited resources. There was a constant and stable increase of followers of 14% on average every quarter and majority of the followers are young adults living with T1D. The increase in number of users over the 12 months has been postulated to be driven by several factors. Firstly, the launch of the website in each country using social media, posters and awareness raised from healthcare professionals were likely to have raised the interest and curiosity to discover the website after a country’s launch activities. Targeted webinars for healthcare professionals such as carb counting webinars also drove increase of users in other countries. Analytics also show that healthcare professionals are not often browsing for content but interacting with a specific event which is very different from the expected behavior of a T1D or a caregivers.

It is noted that many patients travel very far to get to the clinic for their appointments and there are very limited resources available in either written leaflets or face to face educational programmes. Furthermore, there are no Diabetes Educators in countries like Cambodia, Laos, Malaysia or Vietnam [1]. A digital programme in local languages to educate and support the T1D population is an initiative that can address the gaps in healthcare for people living with T1D. Plans are underway to launch the Bahasa Malaysia language in March 2023, Burmese language in May 2023, Laotian language in September 2023, and Bahasa Indonesian and Filipino languages in 2024. These digital and novel initiatives in diabetes care and education are crucial in creating a sustainable health capacity that advocates for improving education amongst people living with T1D and their caregivers, provision of medical and psychosocial support, and closing the diabetes healthcare gaps in that continue to exist between developed countries and resource limited countries.

Study limitations include the lack of prevalence and incidence data therefore we do not know the percentage of people living with T1D who are accessing the platform. As A4D is a small charity, we have not undertaken quantitative and qualitative studies on feedback and there is a potential bias on reliance of the data analytics from Google analytics and Facebook analytics to understand the usage of the platform.

Future plans are underway to use the platform to create a local community support and a peer-to-peer networks with online workshops for patients and caregivers, where the community can ask their questions via cha forumst on the Facebook site that can be moderated by patients and caregivers. In addition, we hope that the programmes will be supported by local governments for example in Malaysia, the Hellotype1 in the local language is endorsed by the Ministry of Health.

In the longer term, it is important to advocate for systemic changes in government health policies such as universal coverage of diabetes care, upskilling, education and empowerment among healthcare professionals and people living with T1D to optimise management and improve health and quality of life for people with T1D in SEA. Further research is needed to explore the implementation of this digital educational resource and to consider the impact of this intervention on long-term knowledge retention amongst people living with T1D, their caregivers and healthcare professionals.

## Data Availability

The datasets used and/or analysed during the current study available from the corresponding author on reasonable request.
